# A Radiological Assessment of the Prevalence of Osteoporosis in Male Patients Seen in a South African Hospital: A Retrospective Analysis

**DOI:** 10.1155/2022/1238927

**Published:** 2022-05-04

**Authors:** Lebohang Siwela, Nausheen Khan, Adziambei Mudau

**Affiliations:** Department of Radiology, Faculty of Health Sciences, University of Pretoria, Private Bag X20, Hatfield 0028, South Africa

## Abstract

Developing countries are predicted to bear the burden of osteoporosis in the coming decades. The prevalence of osteoporosis in South African men is unknown, but is thought to be rare. Opportunistic screening for osteoporosis can be performed using quantitative computed tomography (CT) obtained for various clinical indications. We assessed the frequency of osteoporosis in male patients using quantitative computed tomography (CT) obtained for various clinical indications. Data were collected from abdominal and spinal CT scans performed at the radiology department of a provincial tertiary hospital between January 2019 and January 2021. The CT examinations were derived from 507 male patients (mean age, 45±15 years; 83% Black, 0.8% Coloured, 4.1% Indian and 11.2% White). In the CT scans, the region of interest was placed manually at the axial cross-sections of L1 and L3 vertebrae. Using densitometry, we calculated average bone mass density and T and Z scores. We diagnosed osteoporosis in 18.5% (*n* = 94) of our patients. Only 7.9% of patients younger than 50 had osteoporosis, while 35.9% of patients older than 50 years showed signs of osteoporosis. Osteoporosis was more common amongst White male patients (45.6%) and least common in Black male patients (14.4%). Indian patients had the highest prevalence of osteopenia (42.9%). We successfully used CT scans, obtained for various conditions, to identify large numbers of patients with low bone mineral density (BMD). The prevalence of osteoporosis in this sample is similar to rates reported elsewhere in Africa. Asymptomatic patients at risk of developing insufficiency fractures can be diagnosed and managed early using CT scans, thus preventing unnecessary admissions and reducing osteoporosis-related morbidity and mortality.

## 1. Introduction

The prevalence of osteoporosis among South African men is poorly studied despite many osteoporosis risk factors occurring in South Africa [1]. Awareness of osteoporosis is sorely lacking among men, the population at large as well as health authorities, making it extremely difficult to quantify the burden of the disease [1]. Osteoporosis is defined as a skeletal disorder that is characterized by compromised bone strength, ultimately predisposing a person to an increased risk for fracture [2].

Overall bone strength depends on both quantitative bone mineral density (BMD) and qualitative trabecular microarchitecture [2]. Measuring BMD is the consensus approach to screening and monitoring osteoporosis in populations [2]. Once BMD has been measured, radiologists will calculate the number of standard deviations that the patient's BMD is above or below the mean in the reference population, the T-score [2]. The “Z” score represents the number of standard deviations above or below the mean for age-matched controls [2]. Bone mass density varies according to ethnicity and gender due to differences in bone turnover, body fat composition, diet, socioeconomic status, contraceptive use, load bearing, and lifestyle, including smoking and alcohol consumption [2]. Even though American reference ranges may not be appropriate for the South African population, the World Health Organization (WHO) defines normal BMD as a “T” score greater than −1.0, low bone mass or osteopenia is defined as −1.0 to −2.4, whereas “T” scores equal to or less than −2.5 indicate osteoporosis [2].

Imaging plays an important role in detecting and monitoring osteoporosis, which can substantially reduce osteoporosis-associated morbidity and mortality. Dual-energy X-ray absorptiometry (DEXA) of the central skeleton is considered the gold standard for assessing BMD [3]. In South Africa, DEXA is not readily available in all clinics and various other diagnostic tools are used to determine BMD [4]. Irrespective of which imaging tool is used to measure BMD, the DEXA T-score is still used as the reference standard to diagnose osteoporosis [4].

Several alternative imaging tools are available to measure volumetric BMD, including quantitative computed tomography (QCT) [1]. QCT is a feasible option in resource-limited settings [1]. Due to higher radiation exposure compared to DEXA [2], QCT has been proposed as an opportunistic screening approach that piggybacks on lumbar spine assessments. When piggybacking on other assessments, this screening method requires no additional scanning, radiation, or costs [5].

Furthermore, focusing on a simple ROI (e.g., at L1) requires minimal effort from radiologists, requiring negligible interpretation time. Osteoporosis represents a major public health issue due to aging populations [6], and there is a need for wider screening efforts. In this retrospective, cross-sectional hospital-based study, we assessed the prevalence of osteoporosis, using lumbar spine BMD, in a sample of South African male patients who underwent CT scans performed for various clinical indications.

## 2. Research Methods and Design

### 2.1. Study Design and Setting

The study was a cross-sectional, hospital-based study performed at the Radiology Department of Kalafong Provincial Tertiary Hospital, in Tshwane, South Africa. The study was registered with the National Health Research Database (https://nhrd.health.gov.za/Proposal/Details/100753). The study was reviewed and approved by the University of Pretoria, Faculty of Health Sciences Ethics Committee (388/2021). The need for obtaining signed informed consent was waived for this retrospective analysis. The hospital folder details were converted into a unique number to anonymise the patient.

### 2.2. Study Population

We used nonprobability sampling to select the most recent CT scan. From there, we sampled sequentially backwards until the sample size was reached. We sampled 507 abdominal and spine CT scans obtained between January 2019 and January 2021. Scans were included if they were of South African male patients between the ages of 18 and 75. We excluded scans if they were taken from (1) patients younger than the age of 18, (2) patients without a medical file or no clinical information, (3) patients with a morphologically abnormal lumbar spine (specifically L1 and L3), and (4) patients with a history of spinal surgery.

In this study, the patients self-reported the race and ethnicity, which was noted in their medical files. Reporting of race and ethnicity was guided by Flanagin et al. [7].

### 2.3. Data Collection

Spine and abdominal CT scans were performed on either the Philips Brilliance or Ingenuity multidetector CT (28 and 128 row) scanners (Philips South Africa Health Systems, 54 Maxwell drive, Woodmead, Johannesburg). Both scanners were calibrated daily to ensure the accuracy of CT attenuation values, which correlate well with underlying BMD. Axial images from both scanners were acquired with thin collimation at 120 kVp and reconstructed in spiral mode using 2 mm slice thickness for abdominal scans and 1 mm slice thickness for spinal scans with an in-plane resolution of 0.8 mm.

**Figure 1:**
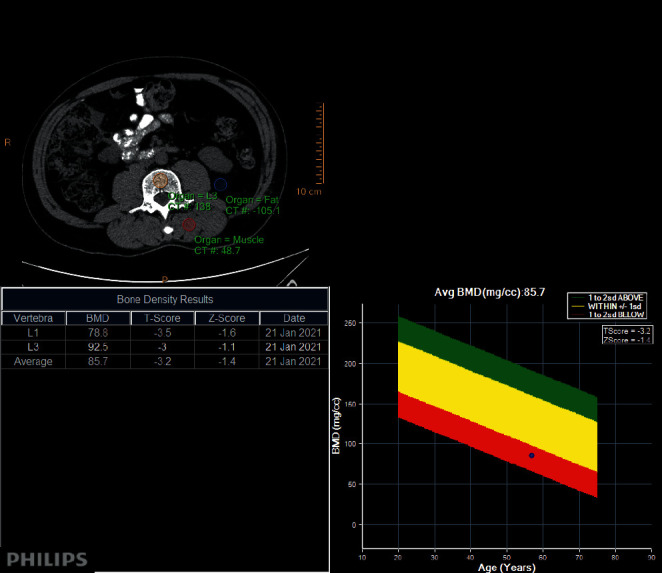
Philips bone mineral density application. An anonymised male patient selected from the PACS database at Kalafong Provincial Tertiary Hospital analysed on ISP using the bone mineral analysis application. The result shows a patient with osteoporosis (bone mineral density of 85.7, T score −3.2, and Z score −1.4).

Once we accessed the picture archiving and communications system (PACS), we filtered scans using descriptive and demographic criteria. The axial CT scans were retrospectively analysed on a standard radiology PACS workstation, using the Philips ISP (Intelli Space Portal) CT bone mineral analysis application. In this phantomless application, the patient serves as a reference as it uses paraspinal muscle and subcutaneous fat as calibration references [8]. This system yields highly reproducible results and avoids beam hardening and scatter effects caused by an external phantom [8]. In comparison to a phantom-based system, this phantomless application is accurate and precise for the diagnosis of lowered bone mineral density [8]. We viewed images using soft tissue and bone windows which do not influence attenuation or bone mineral density values. Mean CT attenuation was assessed by focusing on the ROI at the axial cross-sections of L1 and L3 vertebral bodies. Using the ISP densitometry application, we calculated average BMD and T and Z scores as shown in Figure 1. We avoided placing the ROI over areas of attenuation heterogeneity, such as the posterior venous plexus, spinal haemangiomas, spinal hardware, and compression fractures to avoid distortion of attenuation measurements. The outcome variables were then captured for each patient.

Patients were classified as having osteoporosis if their computed tomography Hounsfield units (HU) in the ROI corresponded to a T-score of −2.5. In the ROI, T-scores equaled −2.5 if L1 ≤110 HU or L3 ≤85 HU [9]. These values are 88.5% specific and 60.8% sensitive for distinguishing osteoporotic lumbar spine from nonosteoporotic lumbar spine [9].

### 2.4. Data Analysis

The data were analysed using the “SAS institute” data management software (version 9.4). The data were analysed using descriptive statistics and categorical and continuous variables. Pearson chi-square test was used to test the association between BMD and age. Fisher–Freeman–Halton exact test was used to investigate the association between BMD and race.

## 3. Results

### 3.1. Demographic Information

Of the 507 scans included, the mean age (± standard deviation) of patients was 45.15 ± 14.79 years. The median age was 43 years and ranged from 20 to 75 years. Most scans were from Black patients (*n* = 425, 83%) followed by White (*n* = 57, 11.2%), Indian (*n* = 21, 4.1%), and Coloured (*n* = 4, 0.8%) patients.

### 3.2. Prevalence of Osteoporosis

**Figure 2:**
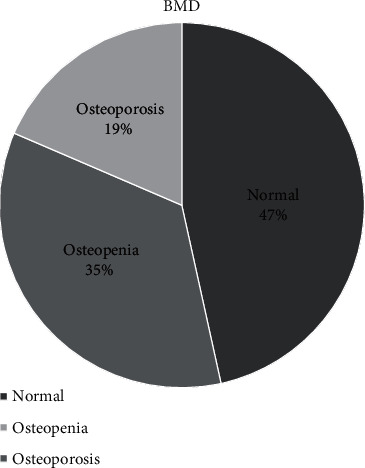
Prevalence of osteoporosis and osteopenia amongst male patients (*n* = 507) who underwent CT scans at Kalafong Hospital, South Africa.

In our sample, 18.5% (94/507) of patients showed signs of osteoporosis and 34.9% (177/507) indicated osteopenia (Figure 2).

### 3.3. Bone Mineral Density and Age

Overall, of the 507 patients, 315 (62.1%) were younger than 50 and the remaining 192 (37.9%) were older than 50 years. Young men had the highest lumbar spine BMD which decreased progressively with age. Young (<50 years, 35.6%) and older (>50 years, 33.9%) patients had similar levels of osteopenia. Younger (<50 years) patients had a lower (7.9%) rate of osteoporosis than older (>50 years) patients (35.9%).

### 3.4. Bone Mineral Density and Race

**Figure 3:**
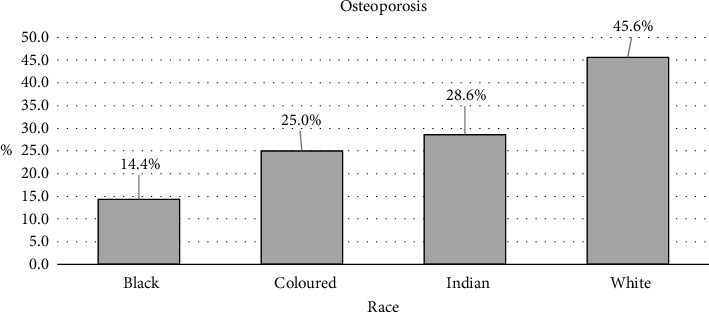
Proportion of patients by racial group, diagnosed with osteoporosis identified from opportunistic CT scans of men at Kalafong Hospital, Tshwane, South Africa.

Figure 3 shows the prevalence of osteoporosis within each racial group. The prevalence of osteoporosis was highest among White male patients (45.6%) and lowest in Black male patients (14.4%). Indian male patients had the highest prevalence of osteopenia (42.9%).

## 4. Discussion

In this study of 507 South African male patients, we show the clinical utility and feasibility of using opportunistic abdominal and spine CT scans to measure lumbar spine BMD, to screen for osteoporosis. In our sample, 18.5% of male patients were diagnosed with osteoporosis, which is similar to the global prevalence of osteoporosis of 18.3%, for men and women based on 86 studies across five continents [10]. In Africa, the prevalence of osteoporosis may be much worse than on other continents [10]. In a prevalence study conducted in Egypt, osteoporosis was estimated to affect 21.9% of men [1]. Currently, osteoporosis is estimated to affect 11.7% of men, globally [10], which is much lower than reported in our study. The prevalence of osteoporosis in South Africa remains unknown, but best estimates suggest that the prevalence of osteoporosis in the lumbar region is.

The prevalence of osteoporosis in South Africa remains unknown, but best estimates suggest that the prevalence of osteoporosis in the lumbar region is 16% and 24% in Ward's area of the femoral neck, respectively, [11].

In our study, younger men had higher BMD and lower prevalence of osteoporosis, whilst older men had lower BMD and a higher prevalence of osteoporosis. In people, the prevalence of osteoporosis is expected to increase with advancing age which is generally secondary to declining bone quality [12]. Trabecular bone loss in men occurs in the third decade and is a result of trabecular thinning due to the gradual decline of bioavailable testosterone [12]. Other studies in men older than 50 years have reported a lower prevalence of osteoporosis. In Morocco, an estimated 13.4% of men aged 50 years and older have osteoporosis [1], far fewer than reported in our study. In another study, the prevalence of osteoporosis in men older than 50 years was found to be between 3 and 6% when using a male normotensive database [12].

Male osteoporosis is an increasingly important public health problem. From age 50 onwards, one in three osteoporotic fractures occurs in men and fracture-related morbidity and mortality are even higher than in women [12]. The lifetime risk of a 50-year-old man for osteoporotic fracture is 13%–25% [13]. In men, the hip, vertebrae, forearm, and humerus are the most common sites affected [13]. About 20% of hip fractures occur in men [13]. By 2050, the worldwide incidence of hip fracture in men is projected to increase by 310% [13]. In addition, 1 out of 8 men older than 50 years will incur an osteoporotic-type fracture during his lifetime, with roughly 30% being hip fractures [14]. Life expectancy in Africa has increased significantly in the last two decades. The population of people aged 60 years and over is expected to triple across the continent, from 46 million in 2015 to 147 million by the year 2050 [1]. The combination of an expected increase in the aged population, rapid urbanisation, and changes in secular trends in Africa is anticipated to lead to a proportional rise in noncommunicable diseases (NCD) including osteoporosis [1].

Ethnic differences in the BMD and fracture risk are well recognized [15]. Studies on the African continent reveal people of African ancestry have a lower incidence of fragility fracture than Caucasians [15]. In relation to race, there is a perception that osteoporosis and its consequent fragility fractures are rare amongst Black people [1]. In our study, the prevalence of osteoporosis among Black male patients was 14.4%, the lowest compared to other racial groups studied. The racial differences noticed in our study, require further study at the population level to uncover predisposing factors. In our study, 42.9% of Indian patients had low bone mass density or osteopenia. This may be related to vitamin D deficiency. It is well known that vitamin D deficiency is common in Africa, with approximately 50–90% of persons on the continent having hypovitaminosis D [1]. Vitamin D plays a pivotal role in regulating and metabolising calcium and phosphorus, which are important for bone formation and mineralization. Vitamin D deficiency is associated with low bone mass and osteoporosis [1]. In South Africa, the prevalence of vitamin D deficiency (≤30 nmol/L) was found to be 3.0% in the Black and 15.0% in the Indian population [1].

The opportunistic screening of osteoporosis using abdominal and spine CT scans is clinically feasible and requires no additional cost or exposure to ionizing radiation. This approach is relevant in South Africa because access to CT is greater than access to DEXA. As noted in this study, we assessed BMD and osteoporosis without additional equipment or patient time, suggesting that this approach is suitable for opportunistic screening of osteoporosis, and also to establish the national prevalence of osteoporosis, specifically in men.

In this study, we report on the rates of osteoporosis in a sample of male patients from South Africa. Our findings cannot be generalised to the whole of South Africa. Firstly, the men in our study were patients seen at a single tertiary hospital set in an urban area of Pretoria. Secondly, our sample included a larger sample of younger men. Thirdly, we did not evaluate modifiable risk factors such as smoking, alcohol consumption, human immunodeficiency virus (HIV), and glucocorticoid therapy.

In conclusion, we show that using CT scans is a feasible tool for osteoporosis screening. We identified that almost 20% of men may suffer from osteoporosis, with higher rates in older men. Future studies should enumerate the prevalence of osteoporosis amongst the whole South African population of men, with the aim of preventing fractures, preventing unnecessary admissions, reducing osteoporosis-related morbidity and mortality, and supporting the national health economic analyses.

## Data Availability

The datasets are available through the corresponding author upon reasonable request.
